# Phase II study of pazopanib in combination with paclitaxel in patients with metastatic melanoma

**DOI:** 10.1007/s00280-018-3624-6

**Published:** 2018-06-25

**Authors:** John P. Fruehauf, Monica El-Masry, Katherine Osann, Basmina Parmakhtiar, Maki Yamamoto, James G. Jakowatz

**Affiliations:** 1 Division of Hematology/Oncology, Chao Family Comprehensive Cancer Center, University of California, 101 The City Drive South, Bldg 56, Orange, Irvine, CA 92868 USA; 2 Division of Surgical Oncology, Chao Family Comprehensive Cancer Center, University of California, 101 The City Drive South, Bldg 56, Orange, Irvine, CA 92868 USA

**Keywords:** Pazopanib, Metronomic paclitaxel, Metastatic melanoma, Antiangiogenic agent, BRAF

## Abstract

**Purpose:**

This phase II study evaluated the safety and clinical activity of pazopanib, a potent and mutlitargeted tyrosine kinase inhibitor (TKI) of vascular endothelial growth factor receptors (VEGFRs)-1, -2 and -3, platelet-derived growth factor receptor (PDGFR)-α and β, and cKit, in combination with metronomic paclitaxel in patients with metastatic melanoma.

**Experimental design:**

Sixty chemotherapy-naive patients received pazopanib at a starting dose of 800 mg daily in combination with metronomic dosing of paclitaxel 80 mg/m^2^ weekly thrice every 4 weeks. The primary endpoint was 6-month progression-free survival (PFS) rate, while secondary endpoints included 1-year overall survival rate, RECIST response rates, progression-free survival rates and median overall survival. Prior BRAF-targeted therapy or checkpoint inhibitors were permitted.

**Results:**

The 6-month PFS rate was 68%, with a 1-year OS rate of 48%. Objective response rate was 37% comprising one complete and 20 partial responses. Stable disease at 8 weeks was noted in 32 patients (55%) with an overall clinical benefit rate of 93%. Six-month median progression-free survival was 8 months and median OS was 12.7 months. The most frequently (> 15%) reported non-hematologic, treatment-related adverse events were fatigue, diarrhea, hypertension, transaminitis and peripheral neuropathy. Treatment-related non-fatal bowel perforation, a known class effect, occurred in one patient. No significant association was noted between plasma levels of pazopanib and response.

**Conclusions:**

The combination of pazopanib and metronomic paclitaxel was well-tolerated, demonstrating significant activity in metastatic melanoma. Further evaluation of this combination is warranted.

## Introduction

The National Cancer Institute estimates that 87,110 new cases of melanoma will be diagnosed and 9730 deaths from the disease will occur in the United States in 2017 [1]. While surgery is often curative with early-stage melanoma, metastatic melanoma has had a median survival time of only 6–9 months [2]. However, the treatment paradigm for patients with stage IV melanoma has changed in recent years leading to improved survival.

Current therapies for advanced melanoma typically include BRAF (BRaf proto-oncogene, serine/threonine kinase) and MEK (mitogen-activated protein kinase)-targeted agents and/or checkpoint-targeted immunotherapy (ipilimumab, pembrolizumab, and nivolumab). Both approaches have been shown to extend survival [3]. Combinations of BRAF and MEK inhibitors, dabrafenib plus tramietinib and vemurafeniib plus cobimetinib, have demonstrated 60–70% response rates, median PFS of 11 months and median OS of 25.1 months [4–6].

With respect to the checkpoint inhibitors, ipilimumab which targets T cell CTLA-4, was the first agent in this class to show an improved OS in melanoma, and meta-analysis of pooled data from ipilimumab trials which included 1861 melanoma patients revealed a 3-year OS rate of 22% [7, 8]. The PD-1 targeted agents pembrolizumab and nivolumab were subsequently shown to be effective in melanoma [3].

In spite of the great advance offered by BRAF/MEK and checkpoint inhibitors, PFS remains below 1 year for both classes of therapy and median OS is approximately 24 months. There is an unmet need for a second- or third-line therapy that could offer further incremental improvements in PFS and OS.

Tumor angiogenesis, mediated by the vascular endothelial growth factor (VEGF) signaling network, is strongly implicated in melanoma progression [9–11]. In patients with melanoma, elevated levels of VEGF are associated with poor outcome [12]. Additionally, preclinical studies have shown that simultaneous inhibition of VEGF receptors (VEGFRs)-1 and -2, but not the sole inhibition of either receptor, blocked melanoma growth and metastasis [13]. Taken together, these findings strongly suggest the involvement of VEGFR signaling pathways in melanoma and support the hypothesis that VEGF-targeted antiangiogenesis therapy (AAT) may prove to be effective either alone or in combination with other therapies.

The benefits of AAT have been shown in two phase II trials for advanced-stage melanoma [14, 15]. The first trial evaluated the activity of axitinib, a selective inhibitor of VEGR-1, -2 and -3 [14]. For the 32 patients enrolled, the objective response rate (RR) was 18.8%, comprising one complete response and five partial responses with a median response duration of 5.9 months (95% CI, 5.0–17.0). Stable disease at 16 weeks was noted in six patients (18.8%), with an overall clinical benefit rate of 37.5%. Six-month progression-free survival (PFS) was 33.9%, 1-year overall survival was 28.1%, and median overall survival was 6.6 months. While these data suggested moderate single-agent activity, combining antiangiogenesis agents with chemotherapy was hypothesized to be more effective. The combination of carboplatin and paclitaxel (CP) alone was subsequently evaluated versus CP plus bevacizumab (CPB) [15]. Two hundred fourteen patients (73% with M1c disease) were randomly assigned. With a median follow-up of 13 months, median PFS was 4.2 months for the CP arm and 5.6 months for the CPB arm (HR, 0.78; *P* = .1414). Overall RRs were 16.4 and 25.5%, respectively (*P* = .1577). With 17-month follow-up, median OS was 9.2 versus 12.3 months, respectively (HR, 0.79; *P* = .1916). No new safety signals were observed.

These results indicated that AAT was active in advanced melanoma and that it could be combined with chemotherapy. We, therefore, designed and initiated a phase II trial evaluating daily oral pazopanib, a potent and mutlitargeted TKI of vascular endothelial growth factor receptors (VEGFRs)-1, -2, and -3, in combination with paclitaxel in patients with metastatic melanoma [16]. This combination was selected based in part on favorable phase I safety and pharmacokinetic data [17]. We employed a metronomic schedule for paclitaxel based on preclinical and clinical data indicating that metronomic administration enhances paclitaxel’s antiangiogenesis effects [18–20].

## Materials and methods

### Patients

Patients ≥ 18 years of age with histologically confirmed advanced melanoma who were chemotherapy-naive were eligible for enrollment. Previous cytokine, immunotherapy or BRAF-targeted therapy was permitted, but had to be completed 28 days prior to first dose of study medication. Other eligibility criteria included measurable disease based on Response Evaluation Criteria in Solid Tumors (RECIST), adequate major organ function, Eastern Cooperative Oncology Group performance status of 0 or 1, and informed consent. Patients were excluded if they met any of the following criteria: previous treatment with antiangiogenic agents, preexisting uncontrolled hypertension, i.e., systolic blood pressure (BP) > 150 mm Hg and diastolic BP (dBP) > 90 mm Hg, active seizure disorder, and major surgical procedure or radiation therapy within 4 weeks of treatment. Prior central nervous system (CNS) metastases was allowed for subjects who had previously treated CNS metastases (surgery ± radiotherapy, radiosurgery, or gamma knife) and were asymptomatic, had no clinical evidence of active CNS metastases for ≥ 28 days prior to enrollment and had no requirement for steroids or enzyme-inducing anticonvulsants (EIAC). Patients with a recent history (6 months) of myocardial infarction or myocardial disease requiring stenting or angioplasty, or heart failure associated with ejection fractions below 50% were excluded.

### Study design

This was an open-label, phase II trial of the clinical activity, safety, and tolerability of pazopanib plus metronomic paclitaxel in patients with unresectable stage III or metastatic melanoma. The primary objective was to determine the 6-month progression-free survival (PFS) rate. Secondary endpoints included the 1- and 2-year survival rates, median progression-free survival, median overall survival, objective response rate, and clinical benefit rate (SD + PR + CR). This study was approved by the institutional review board and was carried out in accordance with the International Conference on Harmonization Good Clinical Practice guidelines protocol. Written informed consent was obtained prior to patients entering the study. The study was registered at ClinicalTrials.gov (NCT01107665).

### Study treatment

All subjects enrolled received pazopanib 800 mg daily dosing in combination with a metronomic dose of paclitaxel 80 mg/m^2^ weekly for 3 weeks every 4 weeks. Subjects were permitted to receive full supportive care during the study, including transfusion of blood and blood products, treatment with antibiotics, anti-emetics, anti-diarrheal agents, analgesics, erythropoietin, colony stimulating factors or bisphosphonates when appropriate.

Study treatment continued until subjects experienced disease progression, death, unacceptable toxicity, or withdrawal of consent for any other reasons. Treatment was interrupted in patients with AE grade ≥ 2 that was not controlled by supportive medication and was resumed at the same dose after resolution to grade 1 or baseline levels. Treatment was resumed at a 20% lower dose after resolution to grade 1 or baseline levels for non-hematologic AEs grade ≥ 3, grade 4 hematologic AEs, or recurrent subjectively intolerable toxicity. At each visit during the treatment period, subjects were evaluated for the occurrence of AEs and laboratory abnormalities. Dose adjustments were implemented whenever clinically indicated. If dose reduction was necessary, two dose reductions for pazopanib were permitted in a stepwise fashion (initially to 600 mg and subsequently to 400 mg if necessary) to achieve resolution of toxicity to grade 1 or baseline. If the toxicity did not recur or worsen, the dose was then increased stepwise back to 600 and 800 mg after monitoring for 10–14 days at each step if toxicity did not recur or worsen. Dose interruptions also occurred for uncontrolled elevated BP, hemoptysis, or proteinuria.

For patients experiencing ≥ grade 3 toxicity attributed to paclitaxel, an initial dose reduction to 65 mg/m^2^ was implemented. Upon recovery, the paclitaxel dose could be re-escalated to 80 mg/m^2^ weekly at the discretion of the investigator. Subjects requiring frequent omissions or dose reductions for individual infusions (eg, one-half of administered doses during a two-cycle period), were allowed to discontinue treatment with paclitaxel and resume therapy with pazopanib monotherapy per investigator discretion. Patients deriving clinical benefit could continue to receive treatment after meeting criteria for study completion.

### Study assessments

Baseline screening with CT CAP and MRI of the brain, ECHO cardiograms and ECG was required. WB PET/CT could be carried out in lieu of CT CAP. Tumors were measured using computed tomography (CT) or magnetic resonance imaging at least every 8 weeks. RECIST 1.1 was utilized for response determinations. Blood samples were collected on day 1 (pre-dose) and every 4 weeks thereafter for analysis of blood counts, complete metabolic profile including LDH. A thyroid panel, urine spot creatinine and protein as well as amylase and lipase were monitored every 8 weeks.

### Analysis of blood-based pazopanib

Plasma samples for assay of serum pazopanib levels were collected C1D1 (pre-dose), C2D1 and C2D28.

### Statistical methods

The study was conducted using a two-stage Simon Minimax design [21]. Due to lower response rates to conventional chemotherapy for this indication, the p0 and p1 were set at 5 and 20%, respectively. The *α* and *β* error rates were set at 0.10 and 0.10, respectively. These criteria resulted in a sample size of 18 patients in stage 1 and a minimum of 14 patients in stage 2 (based on Power Analysis and Sample Size 2002 software, Kaysville, UT). At least one confirmed response (i.e., PR or CR) was needed in stage 1 to allow expansion of the trial to stage 2. Safety and efficacy analyses included all patients who received at least one dose of pazopanib and paclitaxel and had a baseline assessment of disease. Patients who died, progressed, or discontinued treatment prior to experiencing a CR or PR were classified as non-responders. An analysis for constructing the historical control 6-month PFS rate (performance status, presence of visceral disease, brain metastases and gender) was performed based on the calculation method described by Korn et al. [22]. Statistical analysis was performed using GraphPad Prism, GraphPad Software, Inc.

## Results

### Patient characteristics

60 patients were enrolled in the study and received at least one dose of pazopanib and paclitaxel. Patient baseline characteristics are summarized in Table 1. Two patients had unresectable stage III disease, while 58 were stage IV. Twenty-three patients (38%) had received prior systemic treatment for any disease stage. Eleven patients (18%) had prior checkpoint inhibitor treatment, while only four patients (7%) had prior BRAF-targeted therapy. One patient had received adjuvant temozolomide. Lung and liver were the most common metastatic disease sites. CNS involvement was present at baseline in eight patients (13%). Thirty-five patients (58%) were classified as M1C at baseline.

**Table 1:** (a) Patient characteristics at baseline and (b) prior therapy

	Pazopanib (*N* = 60)
(a)	
Median age, years	64
Range	36–90
Sex, *n* (%)	
Male	45 (75)
Female	15 (25)
ECOG performance status, *n* (%)	
0	49 (82)
1	11 (18)
Metastatic stage, *n* (%)	
III	2 (3)
M1A	6 (10)
M1B	17 (28)
M1C	35 (58)
Common metastatic sites,* n* (%)	
Lung	29 (48)
Liver	12 (20)
CNS	8 (13)
BRAF mutant positive	14 (23)
(b)	
Prior therapy, *n* (%)	
Any	23 (38)
HD IFN	11 (18)
Ipilimumab	9 (15)
Pembrolizumab	2 (3)
BRAF inhibitor	4 (7)
Vaccine	2 (3)

The median duration of treatment was 10.6 months (range, 0.4–42.3) with 20 patients (33%) receiving therapy for ≥ 12 months. Treatment discontinuation was related to adverse events/complications in 6 (10%) patients, death on study in 1 patient (1.7%), disease progression during active treatment in 43 (72%), discontinuation of treatment for other complicating disease state 3 (5%), patient withdrawal or refusal after beginning protocol therapy 5 (8.3%) and treatment completed per protocol criteria 1 (1.7%). One patient remains on active treatment with pazopanib and paclitaxel (1.7%).

### Clinical activity

**Fig. 1:**
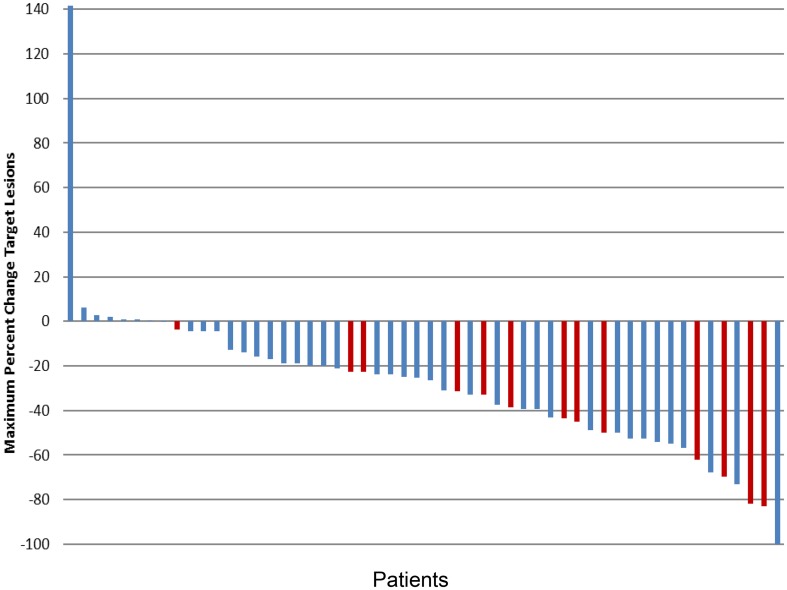
Maximum percentage change in target lesion size, based on response evaluation criteria 1.1 in solid tumors (*n* = 54; six were invaluable for response). BRAF mutation-positive cases shown in red bars

**Fig. 2:**
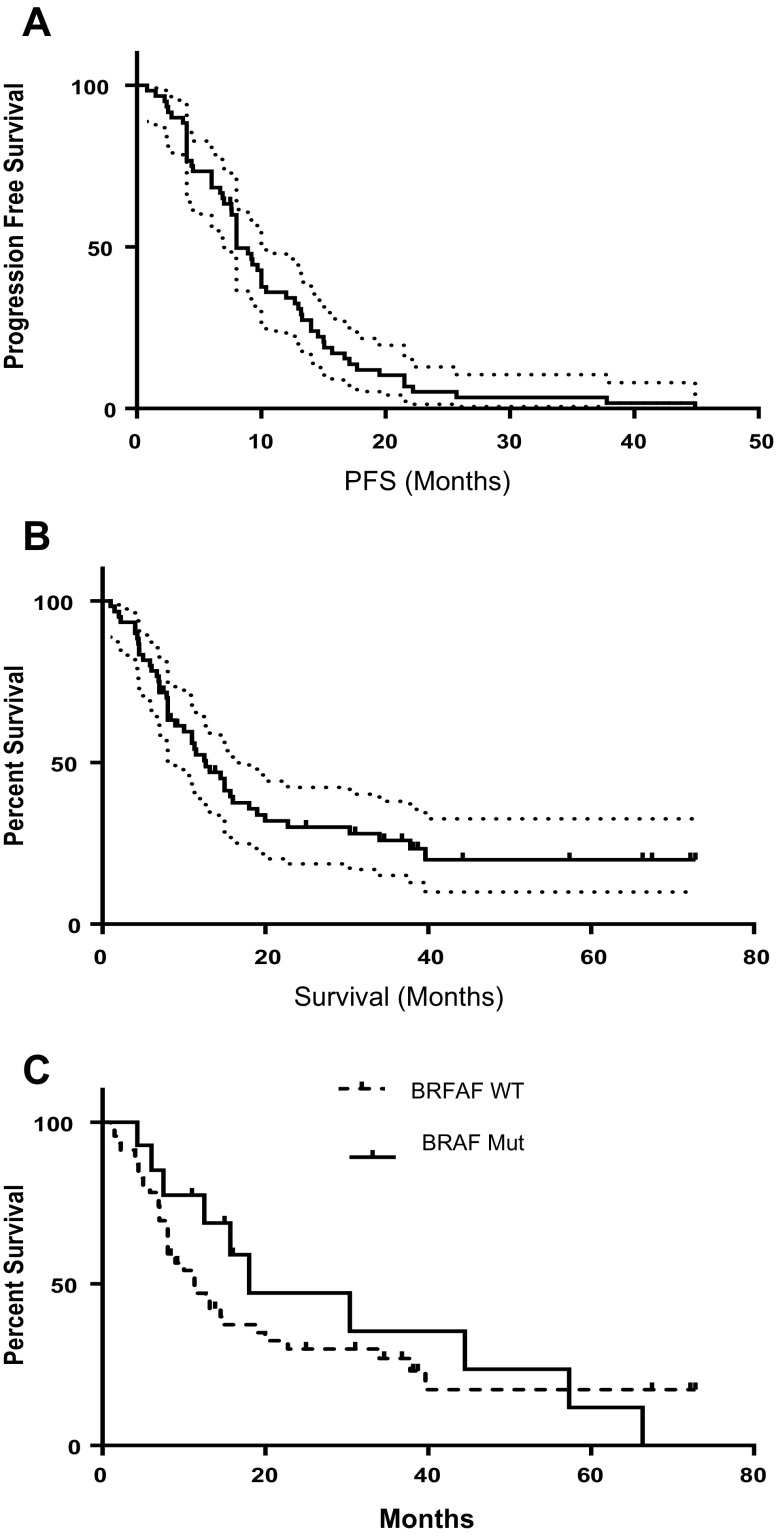
Kaplan–Meier estimates (± 95% CI) of **a** progression-free survival in all patients (*n* = 60); and **b** overall survival (*n* = 60); and **c** comparison of OS between BRAF WT/unknown (*n* = 46) and BRAF mutation-positive patients (*n* = 14)

The ORR was 37% comprising one CR (2%) and twenty PRs (34%) (Table 2). The maximum percentage change in target lesion size is shown in Fig. 1. Thirty-two patients (55%) had a best response of stable disease at least 8 weeks in duration, yielding an overall clinical benefit rate (percentage of patients with a best response ≥ stable disease) of 91%. An additional four patients (7%) had progressive disease. Median PFS was 8 months (95% CI shown on Kaplan Myer Plot) (Fig. 2a), and median OS was 12.7 months (95% CI shown on Kaplan Myer Plot) (Fig. 2b). Six-month PFS was 68% and 1-year OS was 48.1% and 2-year OS was 27%. The response profile for the BRAF WT subset (*n* = 44) was 1 (2%) CR, 12 (27%) PR, 28 (64%) SD and 3 (7%) PD. For the BRAF V600E-positive cases (*n* = 14) there were 8 (57%) PR, 5 (36%) SD and 1 (7%) PD. While there was a trend towards improved OS in the BRAF mutant subset (18 months) versus the BRAF WT/unknown group (11.3 months), this was not statistically significant (HR 0.784 95% CI 0.41–1.54, Log Rank *P* = .49).

### Safety

The most frequently (> 15%) reported non-hematologic, treatment-related AEs included fatigue, hypertension, hoarseness, and diarrhea, (Table 3). The majority of these events were grade 1/2. The most common grade ≥ 3 AE was transaminitis (*n* = 20; 33%) followed by fatigue (*n* = 8; 13%) and hypertension (*n* = 8; 13%). There was one case of bowel perforation requiring surgery (*n* = 1; 1.7%). There were 9 patients who had dose reductions in pazopanib 9 (15%), 42 (70%) who had treatment interrupted, and therapy discontinued in 10 (16.7%) cases.

**Table 2:** Response rates *N* = 58 evaluable

ORR	21 (36%)
DCR (CR + PR + SD)	53 (91%)
CR	1 (2%)
PR	20 (34%)
SD	32 (55%)
PD	4 (7%)

**Table 3:** Safety findings: non-hematologic, treatment-related adverse events (AEs) reported by at least 15% of patients or of clinical interest, and hematologic abnormalities

	Number of patients, *N* = 32 *n* (%)	
	Total^a^	Grade 3/4^a^
Non-hematologic AEs		
Fatigue	44 (73)	8 (13)
Diarrhea^b^	37 (62)	3 (5)
Hypertension^b^	35 (48)	1 (2)
Nausea	29 (48)	1 (2)
Transaminitis	21 (35)	10 (17)
Hematologic abnormalities^c^		
Anemia	13 (22)	0
Thrombocytopenia	4 (7)	0
Neutropenia	18 (30)	9 (15)

^**a**^National Cancer Institute Common Terminology Criteria for Adverse Events, version 3.0

^b^Not otherwise specified

^c^Based on laboratory data

### PK analysis

**Fig. 3:**
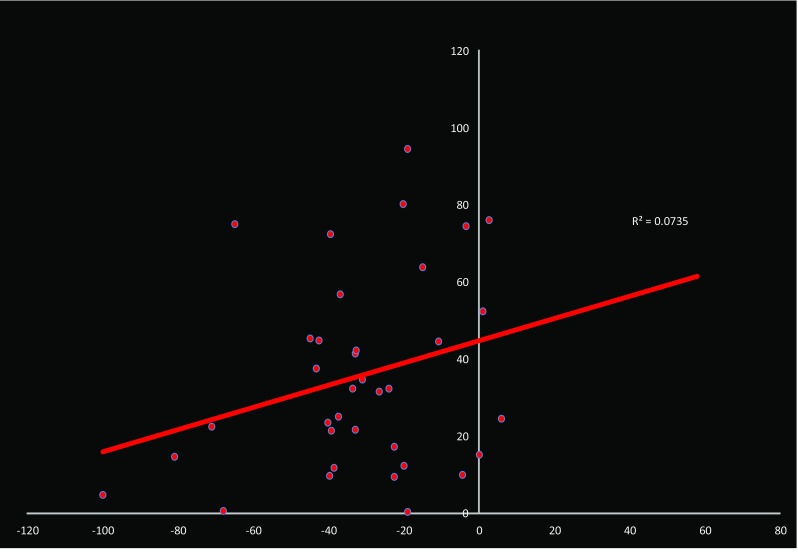
Correlation coefficient between pazopanib pre-dose blood levels on C2D1 and maximum percent change in target lesions

Pharmacokinetic analysis of pazopanib blood levels failed to yield a significant relationship with response (Fig. 3). This finding parallels with that seen for axitinib in melanoma [14].

## Discussion

These results demonstrate that pazopanib plus metronomic paclitaxel exhibited significant activity in this group of patients with advanced stage melanoma, of which 58% fell into the poor prognosis stage M1C (Table 1). Antitumor activity was observed with an ORR of 37%, including one CR and twenty PRs, with a median PFS duration of 8 months (Table 3). An additional 32 patients (55%) experienced stable disease lasting at least 8 weeks. Although cross-study comparisons are complicated by methodological differences, this response rate is superior to the 8–13% ORR provided by standard DTIC or temozolomide therapy for advanced disease and exceeds the ORR range of 10–20% associated with interferon-α and IL-2 or carboplatin and paclitaxel combination therapy [23–26]. Additionally, the 6-month PFS rate was 68%, with a 1-year OS rate was 48.1% (Fig. 2). Median OS was 12.7 months (95% CI shown on Kaplan–Meier Plot). No clear association was noted between pazopanib serum levels and response (Fig. 3).

Current first-line therapy for stage IV melanoma generally includes either a checkpoint inhibitor or a BRAF/MEK tyrosine kinase inhibitor combination for patient’s bearing tumors that carry a V600E or V600K BRAF mutation. In the clinical trial that compared the checkpoint inhibitor pembrolizumab to investigator choice chemotherapy (ICC) in patient’s refractory to ipilimumab or to prior BRAF-targeted therapy, pembrolizumab was superior to ICC with respect to PFS and tolerability, providing the basis for accelerated approval in advanced melanoma [27]. In a subsequent head to head comparison, pembrolizumab given every 3 weeks demonstrated greater efficacy than ipilimiumab, with a 6-month PFS rate of 46.4%, and a 12-month survival rate of 68.4% [28]. In updated analysis, pembrolizumab-treated patients had a RR of 36.1%, with a 55.3% 24-month survival rate [29]. These response rate results are similar to those we observed with pazopanib plus paclitaxel, although the 24-month survival rate was lower for pazopanib and paclitaxel at 27%.

Nivolumab, the second PD-1-targeting checkpoint inhibitor approved for melanoma, was compared to investigator choice chemotherapy (ICC) as second- or later-line treatment in patients who were refractory to ipilimumab or BRAF agents. Confirmed objective responses were seen in 31.7 versus 10.6%, respectively [30]. These results were further corroborated in previously untreated patients without a BRAF mutation, where nivolumab was compared to dacarbazine [31]. Patients in the nivolumab arm showed a response rate of 40.0 versus 13.9% in the dacarbazine arm. Nivolumab-treated patients had a median PFS of 5.1 months and a 1-year survival rate of 72.9%. This finding is also similar to the results reported here for pazopanib plus paclitaxel which demonstrated a PFS of 8 months.

The combination of nivolumab with ipilimumab has also been found to be active. The 3-armed phase III study randomized 945 treatment-naive patients with unresectable or metastatic 1:1:1 to nivolumab plus ipilimumab, nivolumab alone, or ipilimumab alone [32]. The combination showed a response rate of 58 versus 44% for nivolumab alone, and 19% for ipilimumab alone. The combination group had a median PFS of 11.5 months. The clinical benefit provided by the combination was most evident in patients with PD-L1-negative tumors. However, the combination arm experienced a high frequency of severe immune-related adverse events with almost all patients reporting at least one side effect, while 57% showed an event of grade 3 or 4. The combination therapy had to be stopped in 39% of all patients due to side effects such as diarrhea with colitis or hepatitis with elevated liver enzymes.

BRAF inhibition can also be considered as first-line therapy for patients with V600E of V600K mutations. Combinations of BRAF and MEK inhibitors, dabrafenib plus trametinib or vemurafeniib plus cobimetinib, have demonstrated 60–70% response rates, median PFS of 11 months and median OS of 25.1 months [4–6]. While the BRAF mutant subset in this trial was limited in size (*n* = 14), the response rate to pazopanib plus paclitaxel was 57% with a median OS of 18 months. Pazopanib does exhibit activity against both WT and V600E mutant BRAF, with an apparent inhibitory concentration of 68 and 160 nM, respectively (Investigator Brochure). Cmax levels of pazopanib are in the order of 130 μM, suggesting that the levels of pazopanib achieved at standard doses can exhibit tyrosine kinase inhibition (TKI) activity against both WT and mutant BRAF. This pharmacodynamic effect of pazopanib may explain the higher RR of 57% we observed for the BRAF subset.

The results of our trial indicate that the pazopanib plus paclitaxel combination can be given safely to patients with advanced melanoma and that its clinical activity is similar to that observed for current first-line treatment. This antiangiogenesis regimen may be of value for patients who fail standard first- and or second-line therapy, offering an additional incremental benefit to patients with advanced melanoma. Further exploration of this regimen is warranted.
